# A case report of Charcot arthropathy caused by syringomyelia and Chiari malformation complicated with scoliosis

**DOI:** 10.1186/1756-0500-7-277

**Published:** 2014-05-02

**Authors:** Heming Liu, Yulu Wang, Zenghua Yang, Kunzheng Wang

**Affiliations:** 1 Orthopedic Surgery of the 2nd Affiliated Hospital of Medical institute of Xi’an Jiaotong University, Xi’an, Shannxi Province, China; 2 No.3 Orthopedic Surgery of the 1st Affiliated Hospital of Baotou Medical College, Baotou, Inner Mongolia Province, China

**Keywords:** Charcot arthritis, Syringomyelia, Chiari malformation, Scoliosis

## Abstract

**Background:**

Although Charcot arthropathy, also known as neuropathic arthropathy, of which early diagnosis and treatment is extremely difficult, associated with other cause factor has been widely described, Charcot arthropathy caused by syringomyelia and Chiari malformation complicated with scoliosis has never been described in the literature.

**Case presentation:**

A 44-year-old male was hospitalized for diagnosis and treatment due to complaining the progressively swelling and limitation of motion in his left shoulder joint for 1 year. The patient has no significant past medical history except for scoliosis 8 years prior to his presentation to our clinic; He denied any constitutional symptoms, trauma, or pain in the upper extremities at this time of presentation. Based on history, physical and auxiliary examination, following diagnoses were made: Charcot arthropathy of the left shoulder, syringomyelia, Chiari malformation and scoliosis.

**Conclusion:**

Once Charcot arthritis was found, it was mostly in advanced stage and very difficult to treat. So we recommended that if patient suffering from scoliosis visited in clinic, further examination such as magnetic resonance imaging (MRI) and regular follow-up should be carried out, and early-stage of this devastating disease caused by syringomyelia and Chiari malformation may be diagnosed easily.

## Background

Charcot arthropathy, also called neuropathic arthropathy, was first described by Jean-Martin Charcot in 1868 [1]. The etiological factor of Charcot arthropathy included tabes dorsalis, syringomyelia, traumatic spinal cord injury, leprosies, congenital pain insensitivity, diabetes mellitus, chronic alcoholic intoxication, and so on. Although Charcot arthropathy associated with other cause factor has been widely described, there were a limited number of case reports describing Charcot arthropathy caused by syringomyelia and Chiari malformation complicated with scoliosis. Thus, we described a patient of this case in this report.

## Case presentation

A 44-year-old male was hospitalized for diagnosis and treatment due to complaining the progressively swelling and limitation of motion in his left shoulder joint for 1 year. Physical examination: the lower thoracic spine presence of scoliosis; the left shoulder apparently swollen, and its motion limited. Moreover, a healed scar by burn was found on the left shoulder of the patient. Both superficial and deep sensations in his left extremities were decreased, and ankle and knee jerks were normal. Pathologic reflex such as Babinski sign was absent.

**Figure 1:**
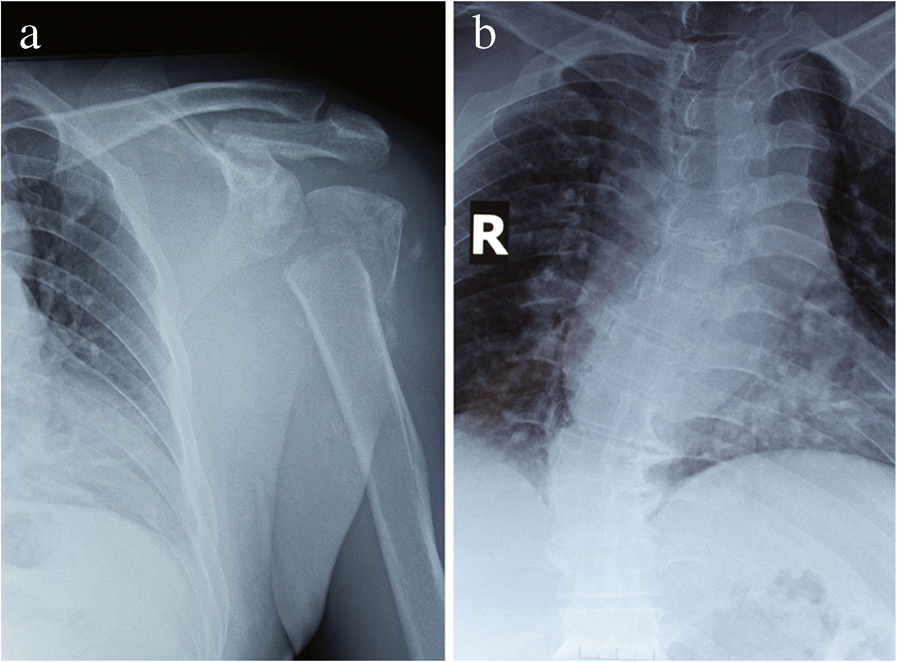
**X-ray of this case.** The surface of articulation of humerus was conspicuous rough, the head of humerus was disappeared completely **(a)**, vertebrate column showed that the right lateral curvature of the lower thoracic vertebra **(b)**.

**Figure 2:**
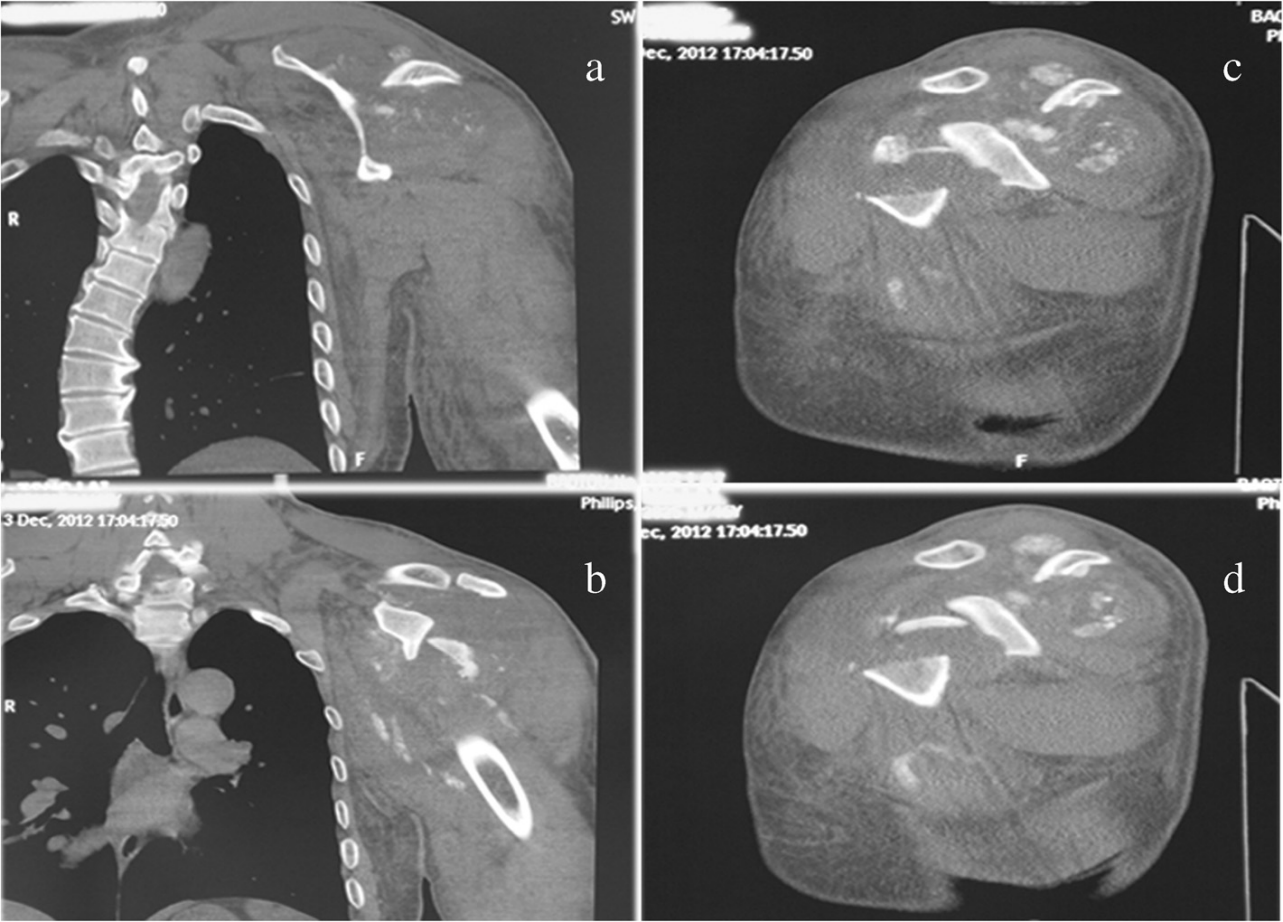
**CT of this case.** The right lateral curvature of the lower thoracic vertebra **(a)**, broken humeral head and sporadic calcification shadow of the surrounding of this joint **(b, c and d)**.

Anteroposterior plain radiograph of the vertebrate column showed that the right lateral curvature of the lower thoracic vertebra was scoliosis. Anteroposterior plain radiograph of the left shoulder revealed an absence of the humeral head because of resorption, soft-tissue swelling, and sporadic calcification shadow near the joint (Figure 1). The same signs were present in the computed tomography (CT) scan of the vertebrate column and left shoulder joint (Figure 2).

**Figure 3:**
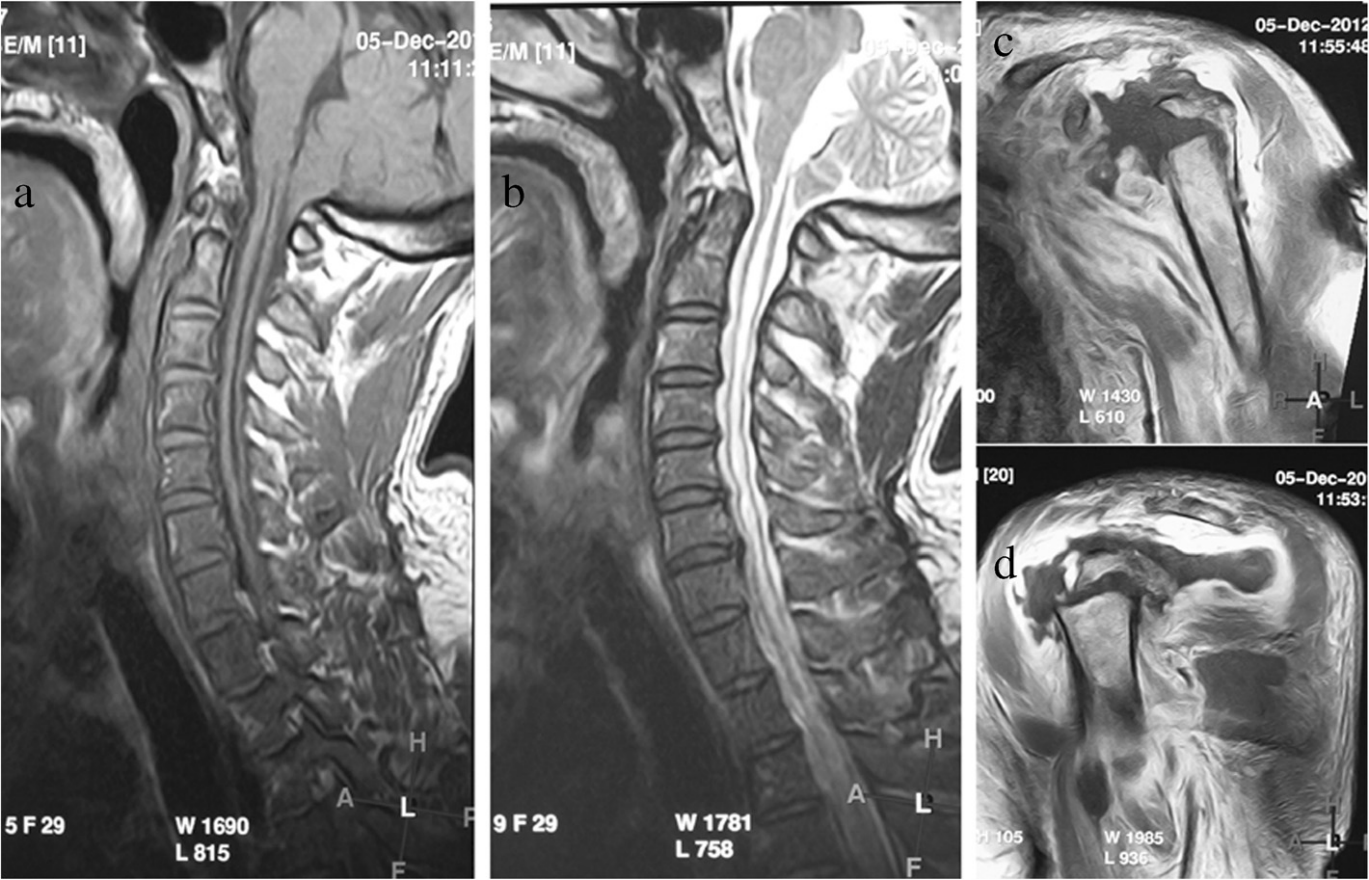
**MRI of this case.** A significant cerebellar tonsillar hernia **(a)**, Syringomyelia from C1 to T4 **(b)**, disappearance of humeral head and swollen soft tissue of the surrounding of the joint **(c and d)**.

A laboratory examinations as well as MRI were ordered. The erythrocyte sedimentation rate was 5 mm/h, the C-reactive protein was 57.6 mg/L, and the leukocyte count was 8.9 × 10^9/L. And acid phosphatase and alkaline phosphatase were normal. The MRI showed a significant cerebellar tonsillar hernia, and the distance of the inferior margin between amygdala cerebelli and foramen magnum was 5 cm. Syringomyelia from C1 to T4 was evidenced and the signal of the fluid in cavity was identical with cerebrospinal fluid (Figure 3). Clinical diagnoses were Charcot arthropathy of the left shoulder, syringomyelia, Chiari malformation and scoliosis.

## Discussion

Chiari malformation was also known as a group of congenital malformations involving the brainstem, cerebellum, upper spinal cord, and surrounding bony structures. Type I is the most common, and characterized by herniation of the cerebellar tonsils through the foramen magnum into the cervical spinal canal. The pathogenesis of Chiari malformation associated with syringomyelia was still a subject of much debate. As follows: (1) The theory of fluid mechanics [2]; (2) The theory of separated pressure of intracranial and spinal canal [3]; (3) The theory of the infiltration of cerebrospinal fluid in parenchyma of spinal cord [4]. Some authors think that the mechanism of Chiari malformation and syringomyelia induced scoliosis may be: syringomyelia damage spinal cord anterior horn and pyramidal tract, caused unbalance of paraspinal muscle, which led to scoliosis [5].

Charcot arthropathy was characterized by the gradual destruction of the affected joint or joints, in patients most with neurosensory loss. Charcot arthropathy is usually seen in weight-bearing joints such as ankle, knee, and hip, while the localization of involved joint changes according to the underlying neurological pathology [6]. Charcot arthropathy occurred most commonly in patients with diabetic neuropathies, and the incidence of diabetic Charcot arthropathy has been reported to range from 0.1% to 5.0% [7]. This destruction happened mostly in foot and ankle [6]. Secondly, Charcot arthropathy can be developed in patients with syringomyelia, and then the shoulder and elbow are the most frequently involved joints in syringomyelia [6]. Charcot arthropathy secondary to syringomyelia mostly involved single joint and involvement of multiple joint is rare [8,9]. Furthermore, the joints mostly involved are the hip and knee in tabes dorsalis [6]; spine in tertiary syphilis, post–spinal cord injury [10], and congenital insensitivity to pain [11]; and tarsometatarsal and subtalar joint in chronic alcohol abuse [12].

The pathogenesis of neuroarthropathy was in debate. Many theories have been proposed to explain the etiology of Charcot arthropathy, including (1) osteopenia owing to increased volume of blood flow associated with autonomic neuropathy; (2) bone failure because of abnormal stress on the bones and joints related to muscle imbalances resulting from sensorimotor neuropathy; (3) joint subluxation due to ligamentous stretching associated with joint effusion; and (4) the loss of protective mechanism in body due to sensory disturbance caused by neuropathy [13,14].

The patient with no significant medical history in the past, except for scoliosis 8 years prior to his presentation to our clinic, visited to our hospital because of complaining the painless swelling and limitation of motion in his joint. He denied any constitutional symptoms, trauma, or pain in the upper extremities at this time of presentation. Then radiographic surveys of the joint were done and the severe destruction in his left shoulder was found. MRI was done and we found that he had syringomyelia complicating with Chiari malformation, which was supposed to be the cause of destruction in his joint.

Once Charcot arthropathy was found, it was mostly in advanced stage and hard for treatment. Although satisfactory middle and long-term results are described in recent reports [15,16], the results of total replacement arthroplasty for the Charcot joints have been disappointing. It is often difficult to distinguish early-stage Charcot arthropathy from other disorders, because no characteristic clinical, radiographic or laboratory finding can confirm the diagnosis of this disease in early stage. As was the case in our report, in his initial visit 8 years ago, there was no examination except for plain X-ray, and the patient had no follow-up visits until 8 years after the initial visit. If MRI had been performed, syringomyelia complicating with Chiari malformation, which was the cause of Charcot arthropathy, would been evidenced. Thus, early diagnosis of Charcot arthropathy would be possibly evidenced. Therefore, we offer this observational case in the hope of sharing some of our experiences with this relatively rare disease, and we recommended that if patient suffering from scoliosis visited in clinic, further examination such as MRI and regular follow-up should be carried out. And early-stage of this devastating disease caused by syringomyelia and Chiari malformation may be diagnosed easily. Perhaps in the future, some of our findings can be used to generate hypotheses for upcoming cohort studies and randomized controlled trials.

## Conclusion

If patient suffering from scoliosis visited in clinic, further examination such as MRI and regular follow-up should be carried out, and early-stage of Charcot arthritis caused by syringomyelia and Chiari malformation may be diagnosed easily.

## Consent

Written informed consent was obtained from the patient for publication of this Case Report and any accompanying images. A copy of the written consent is available for review by the Editor-in-Chief of this journal.

## Abbreviations

MRI: Magnetic resonance imaging; CT: Computed tomography.

## Competing interests

The authors declare that they have no competing interests. No financial support has been received.

## Authors’ contributions

LH: case study, research, and writing of the manuscript. WY, YZ, WK: supervision and review of the manuscript. All authors read and approved the final manuscript.
